# Ovarian cycle-linked plasticity of δ-GABA_A_ receptor subunits in hippocampal interneurons affects γ oscillations *in vivo*

**DOI:** 10.3389/fncel.2014.00222

**Published:** 2014-08-11

**Authors:** Albert M. I. Barth, Isabella Ferando, Istvan Mody

**Affiliations:** ^1^Department of Neurology, The David Geffen School of Medicine, University of California at Los AngelesLos Angeles, CA, USA; ^2^Interdepartmental Graduate Program in Molecular, Cellular, and Integrative Physiology, University of California at Los AngelesLos Angeles, CA, USA; ^3^Department of Physiology, The David Geffen School of Medicine, University of California at Los AngelesLos Angeles, CA, USA

**Keywords:** ovarian cycle, PMS, PMDD, gamma oscillations, GABA_A_ receptor, delta subunit, tonic inhibition, parvalbumin

## Abstract

GABA_A_ receptors containing δ subunits (δ-GABA_A_Rs) are GABA-gated ion channels with extra- and perisynaptic localization, strong sensitivity to neurosteroids (NS), and a high degree of plasticity. In selective brain regions they are expressed on specific principal cells and interneurons (INs), and generate a tonic conductance that controls neuronal excitability and oscillations. Plasticity of δ-GABA_A_Rs in principal cells has been described during states of altered NS synthesis including acute stress, puberty, ovarian cycle, pregnancy and the postpartum period, with direct consequences on neuronal excitability and network dynamics. The defining network events implicated in cognitive function, memory formation and encoding are γ oscillations (30–120 Hz), a well-timed loop of excitation and inhibition between principal cells and PV-expressing INs (PV + INs). The δ-GABA_A_Rs of INs can modify γ oscillations, and a lower expression of δ-GABA_A_Rs on INs during pregnancy alters γ frequency recorded *in vitro*. The ovarian cycle is another physiological event with large fluctuations in NS levels and δ-GABA_A_Rs. Stages of the cycle are paralleled by swings in memory performance, cognitive function, and mood in both humans and rodents. Here we show δ-GABA_A_Rs changes during the mouse ovarian cycle in hippocampal cell types, with enhanced expression during diestrus in principal cells and specific INs. The plasticity of δ-GABA_A_Rs on PV-INs decreases the magnitude of γ oscillations continuously recorded in area CA1 throughout several days *in vivo* during diestrus and increases it during estrus. Such recurring changes in γ magnitude were not observed in non-cycling wild-type (WT) females, cycling females lacking δ-GABA_A_Rs only on PV-INs (PV-*Gabrd*^-/-^), and in male mice during a time course equivalent to the ovarian cycle. Our findings may explain the impaired memory and cognitive performance experienced by women with premenstrual syndrome (PMS) or premenstrual dysphoric disorder (PMDD).

## INTRODUCTION

There are numerous reports about women experiencing fluctuating cognitive and neuropsychological functions during specific stages of the menstrual cycle. During the luteal phase, when progesterone levels are high, some women may experience different levels of dysthymia, irritability, anxiety, impaired working and emotional memory. All of these symptoms are inevitably aggravated in patients with premenstrual dysphoric disorder (PMDD; Man et al., 1999; Sveindóttir and Bäckstrøm, 2000; Bäckström et al., 2003; Reed et al., 2008; Ertman et al., 2011; Rapkin and Akopians, 2012; Yen et al., 2012; Bayer et al., 2014). Although all of these conditions can be hardly ascribed to a single mechanism, ovarian cycle-linked plasticity of δ-GABA_A_Rs and resulting effects on tonic inhibition have been implicated in modifications in anxiety and memory performance in rodents (Maguire et al., 2005; Cushman et al., 2014). Moreover, patients with PMDD seem to be less sensitive to GABAergic modulation during their luteal phase, which led to hypothesize a luteal deficit of GABA_A_Rs plasticity (Bäckström et al., 2003; Maguire et al., 2005).

The δ-GABA_A_Rs are high affinity, low efficacy non-synaptic GABA_A_ receptors with a high sensitivity to neurosteroids (NS; Brickley and Mody, 2012). During times of altered NS levels, δ-GABA_A_Rs expression changes in a direction that seems to depend on the timing of NS fluctuations. For instance, δ-GABA_A_Rs plasticity has been observed during the ovarian cycle, pregnancy, the postpartum, puberty and acute stress, with direct effects on neuronal excitability and network activity. In particular, δ-GABA_A_Rs plasticity has been reported in both principal cells and INs in different rodent brain areas including the hippocampus, different nuclei of the thalamus and the periaqueductal gray (Lovick et al., 2005; Brack and Lovick, 2007; Maguire and Mody, 2007, 2008; Maguire et al., 2009; Ferando and Mody, 2013a; Smith, 2013; MacKenzie and Maguire, 2014).

The tonic conductance mediated by δ-GABA_A_Rs constitutes a powerful constraint over gain of neuronal signal transmission in both principal cells and INs (Mody and Pearce, 2004; Semyanov et al., 2004; Farrant and Nusser, 2005; Walker and Semyanov, 2008; Song et al., 2011). The δ-GABA_A_Rs of hippocampal INs modulate γ oscillations frequency *in vitro* (Mann and Mody, 2010; Ferando and Mody, 2013a). The γ oscillations are a periodic network activity (30–120 Hz) that can be recorded in different brain areas during certain wakefulness states and REM sleep, and arise from a synchronized excitation and inhibition loop between principal cells and PV-INs, which have a critical role in initiating and maintaining local oscillations (Sohal et al., 2009; Wulff et al., 2009; Korotkova et al., 2010; Carlén et al., 2011; Zheng et al., 2011; Lasztoczi and Klausberger, 2014). These oscillations are thought to enable encoding and memory formation in discrete neuronal network, to facilitate spike-time dependent plasticity, and are considered to play an important role in the physiology of learning and memory (Colgin and Moser, 2010; Uhlhaas et al., 2011; Buzsáki and Wang, 2012; Uhlhaas and Singer, 2012). Because in this study we were interested in more gradual alterations in oscillatory activity (expected with the hormonal changes linked to the ovarian cycle) we focused on REM sleep which is characterized by prominent and regular 𝜃 – γ episodes, and is unaffected by instantaneously changing “external” parameters such as running speed (McFarland et al., 1975; Shin and Talnov, 2001; Ahmed and Mehta, 2012); instead, the 𝜃 – γ episodes during REM sleep rely on “internal” mechanisms such as emotional information processing and memory consolidation which are known to be affected by ovarian/menstrual cycle (Montgomery et al., 2008; Walker, 2009; Scheffzük et al., 2011).

In a recent study in mice we showed a homeostatic pregnancy-related down-regulation of δ-GABA_A_Rs in CA3 stratum pyramidale (SP) INs which led to an increase in the frequency of γ oscillations recorded *in vitro* (Ferando and Mody, 2013a), in a manner similar to what has been observed in *Gabrd^-/-^* mice (Mann and Mody, 2010). However, the effects of δ-GABA_A_R plasticity of INs on network activity and dynamics in the intact brain remain to be elucidated. In this study we show ovarian cycle-linked alterations in δ-GABA_A_R expression in hippocampal CA1 and CA3 SP INs, on dentate gyrus granule cells (DGGCs) and pyramidal cells of the CA1, with increased expression during the high progesterone stage of diestrus, and decreased expression in estrus. These changes correlate with periodic modifications in the magnitude of γ oscillations recorded *in vivo* in CA1 SP of freely moving mice.

Previous studies showed increased anxiety and memory performance in female WT but not in global *Gabrd^-/-^* mice during estrus (low progesterone phase), while the behavior during diestrus in WT mice closely resembled that of male mice (Maguire et al., 2005; Moore et al., 2010; van Goethem et al., 2012; Cushman et al., 2014). However, δ-GABA_A_Rs are plastic in both hippocampal principal cells and INs, so that behavioral correlates in global *Gabrd^-/-^* mice cannot distinguish between receptor changes in specific neuronal subtypes. By using a recently engineered floxed-*Gabrd* mouse (Lee and Maguire, 2013) and the PV-IRES-Cre line (JAX Stock # 008069), we specifically deleted the δ dubunits of GABA_A_Rs from PV + INs (PV-*Gabrd^-/-^*) to examine potential changes in ovarian cycle-linked modifications in γ oscillations magnitude in the absence of δ-GABA_A_Rs on PV-INs. Our findings identify a possible underlying cause for the different degrees of cognitive impairment experienced by some women at various phases of the ovarian cycle.

## MATERIALS AND METHODS

### ANIMAL HANDLING

In this study we used adult (15–20 week-old) female and male C57BL/6J mice, WT (*Cre^-/-^*) or mice lacking δ-GABA_A_Rs specifically on PV-INs (PV-*Gabrd^-/-^*), generated by crossing PV-*Cre* (PV-IRES-Cre line; JAX Stock # 008069) and *Gabrd*-flox mice (generous gift of Dr. Jamie Maguire, Tufts University; Lee and Maguire, 2013) both back-crossed for >10 generations on C57BL/6J background. The δ-GABA_A_Rs ablation from PV-IN was confirmed with immunohistochemical fluorescent double labeling (Ferando and Mody, in preparation, data not shown). Mice were housed with *ad libitum* access to food and water and kept on a 12-h light/dark cycle, under the care of the UCLA Division of Laboratory Animal Medicine (DLAM). All experiments were performed during the light period and according to a protocol (ARC # 1995-045-53B) approved by the UCLA Chancellor’s Animal Research Committee. Genotyping was performed by Transnetyx (Memphis, TN, USA).

### SURGERY

Surgeries were performed under aseptic conditions on mice weighing 25–30 g. Under isoflurane anesthesia (2–2.5% in O_2_ alone) the animal was mounted into a standard Stoelting instrument stereotaxic frame with blunt ear bars. Body temperature was maintained at 37^∘^C using a rectal probe and a water circulated heating pad. The cranium was exposed through a small midline scalp incision. The bone was dried and three holes were drilled (0.5 mm diameter) in the cranium. With the aid of a micromanipulator, two sterilized recording electrodes (PlasticsOne, stainless steel, 125 μm diameter) were lowered into hippocampal CA1 region SP, bilaterally (at stereotaxic coordinates: anteroposterior, AP, 5.5 mm; mediolateral, ML, 1.45 mm; dorsoventral, DV, 1.2 mm from brain surface). The third hole was drilled above the cerebellum to insert the ground/reference electrode. The skull surface was covered by thin layer of cyanoacrylate based glue (Insta-Cure+, Bob Smith Industries) and then dental acrylate (Ortho-Jet, Lang Dental Manufacturing Co., Inc.) was used to attach the electrode sockets to the skull surface. Immediately after surgery, the mouse was continuously monitored until recovered, as demonstrated by their ability to maintain sternal recumbency and to exhibit purposeful movement. During the recovery period after surgery, warm saline solution (0.01–0.02 ml/g/twice/day) was administered subcutaneously to prevent dehydration. To prevent any infection around the implant we topically administered Neosporin for 7 days. For analgesia, 0.1 mg/kg of buprenorphine was injected subcutaneously prior to surgery. Buprenorphine injections were continued following the surgery every 12 to 48 h.

### OVARIAN CYCLE INDUCTION AND MONITORING

Female virgin mice are generally anovulatory or have irregular cycles, unless exposed to male pheromones. In the present study ovarian cycle was induced in previously anovulatory virgin adult mice (15–20 week-old) by a single exposure to male bedding, and monitored by means of vaginal impedance measurements and vaginal smears cytological analysis, as previously described (Ramos et al., 2001; Maguire et al., 2005; Jaramillo et al., 2012; Cushman et al., 2014). Briefly, vaginal impedance was measured daily (Estrus cycle monitor EC40, Fine Science Tools). Daily vaginal smears were fixed in methanol and stained (Giemsa Staining, Fisher Diagnostics). Diestrus and estrus were defined as 3 days prior and 1 day after vaginal impedance peak, respectively, and by vaginal cytology profile (e.g., **Figure 5A**). Mice were tested in either diestrus (high plasma progesterone) or estrus (low plasma progesterone) phase of their ovarian cycle.

### *IN VIVO* CHRONIC SIMULTANEOUS VIDEO AND LOCAL FIELD POTENTIAL RECORDINGS

Seven to ten days after the animals had fully recovered from the surgery, chronic simultaneous video and local field potential recordings were carried out continuously (24 h a day) for 1–3 weeks. Video observation was performed through an infrared USB camera mounted on the top of the recording cage. The video was recorded using the open source iSpy software, which calculates in real time the percentage deviation between consecutive frames and generates a text file (activity data) containing time-stamped information on the percentages of frame-to-frame deviation values.

Local field potentials were recorded with a custom-made miniature dual headstage amplifier connected to the electrode sockets mounted on the animal’s head and then wired to an electrical commutator (Dragonfly Inc., or Pinnacle Technology Inc.). The signals from the commutator were fed through a 16-channel extracellular amplifier (A-M Systems model 3500) with a gain of 1,000. Signals were low-pass filtered at 1,000 Hz and sampled at 2,048 s^-1^ per channel, using a National Instruments A/D board.

Continuous data acquisition was carried out using Igor NIDAQ tool (Wavemetrics, Lake Oswego, OR, USA) and data were saved into separate files every week. Activity graphs deriving from the video recordings and corresponding local field potentials were loaded into a custom made software (written in Igor64, Wavemetrics, Lake Oswego, OR, USA) that aligned the two recordings to determine sleep and wake cycles.

### IMMUNOHISTOCHEMISTRY AND MICROSCOPY

Brains were processed and tissue stained as previously described (Ferando and Mody, 2013a). Briefly, mice were transcardially perfused with 4% paraformaldehyde in 0.12 M phosphate buffer, pH 7.3. Fixed brains were sectioned at –16^∘^C with a cryostat (coronal, 35 μm). All sections used for the same analyses (e.g., **Figures 4C,D**) were processed in parallel.

For diaminobenzidine (DAB) δ-GABA_A_Rs stain: quenching of endogenous peroxidases was done in H_2_O_2_ (3% in methanol, 30 min). Slices were blocked in 10% normal goat serum (NGS), 2 h, incubated with anti-δ-GABA_A_R antibody (1:500; generous gift from Dr. Werner Sieghart, Medizinische Universität, Wien, Austria) overnight, then with biotinylated goat anti-rabbit antibody (1:200; Vector Laboratories), 4 h. Amplification was done with HRP-conjugated avidin enzyme complex (ABC Elite; Vector Laboratories), 30 min. Signal was developed with DAB (Vector Lab). All steps were done at room temperature.

Bright field microscopy: digital images were collected with an Axioskop 2 Microscope, AxioCam digital camera system and AxioVision 4.8 software (Zeiss). For the same magnification images were taken under identical conditions of brightness and exposure time. Intensity of labeling was measured as optical density (OD) of the region of interest (ROI) using NIH ImageJ software. For CA1 and CA3 INs the ROI was the soma of all visually identified INs within 30 μm of the pyramidal cell layer, for DGML, CA1 stratum oriens (SO) and radiatum (SR), areas of approximately the same size of identified INs were circled and OD was measured. Reported OD values (represented in arbitrary units, AU) are mean ± SEM (**Table 1**). Statistical significance was determined with the use of statistical tests specified in each section.

**Table 1:** Details of δ-GABA_**A**_Rs expression levels in different cell types of the hippocampus over the ovarian cycle, detected by immunohistochemistry.

ROI or INs	Status	OD (Mean ± SEM; AU)	ROI or INs (*n*)	Sections (*n*)	Mice (*n*)
DGML	Diestrus	157 ± 2	20	6	2
	Estrus	147.7 ± 1.1*	20	6	2
CA1 SO	Diestrus	122.4 ± 1.4	20	6	2
	Estrus	97.8 ± 1.3*	20	6	2
CA1 SR	Diestrus	109 ± 1.6	20	6	2
	Estrus	86.9 ± 1.5*	20	6	2
CA1 SP INs	Diestrus	132.6 ± 1.4	67	6	2
	Estrus	102 ± 1.4*	64	6	2
CA3 SP INs	Diestrus	112.7 ± 2.1	44	6	2
	Estrus	88 ± 1.7*	55	6	2
CA1 SP INs	Diestrus	145.5 ± 1.4	100	8	2
	Estrus	121.5 ± 1.8*	104	8	2
	Males1	149.5 ± 1.2	107	8	2
	Males2	147.6 ± 1.4	109	8	2
	NC females1	155.8 ± 1.2*	114	8	2
	NC females2	177.7 ± 1.6*	113	8	2
CA3 SP INs	Diestrus	119.6 ± 2	63	8	2
	Estrus	84.4 ± 2*	64	8	2
	Males1	120 ± 1.9	63	8	2
	Males2	120.2 ± 2.1	74	8	2
	NC females1	119.7 ± 2.1	59	8	2
	NC females2	124.1 ± 2.5	66	8	2

*Summary by hippocampal region of interest (ROI) or INs of optical density mean values in arbitrary units (AU) ± SEM, and *n*’s for diestrus and estrus in WT cycling females, WT non-cycling females and males. Asterisks denote significance (see **Figure 4** for statistical tests).*

### ELECTROPHYSIOLOGY DATA ANALYSIS

**FIGURE 1:**
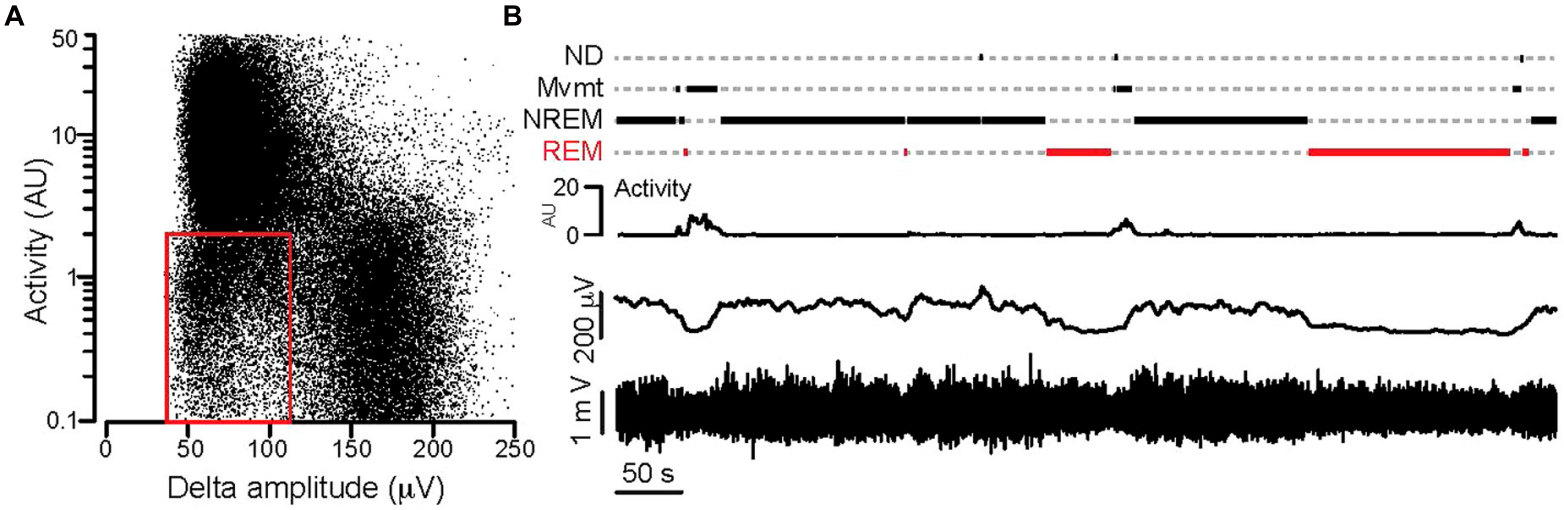
**Separation of behavioral states based on synchronous video and local field potential recordings. (A)** Activity values plotted against the Hilbert magnitudes in the δ frequency range (1–4 Hz) for each 1 s long epoch (total: 86,400 epochs) during a full day of synchronous video-local field potential recording. Note the appearance of three clusters in the point cloud. The *red rectangle* delineates the point cluster corresponding to the putative REM sleep.** (B)** Separation of behavioral states based on combined activity and electrographic thresholds over an ∼12 min period. *Bottom*: local field potential recording, *above*: 1 s binned Hilbert magnitude of the δ-frequency range, *above*: activity graph, *top*: step function showing behavioral states based on activity and δ magnitude values (see text for details; REM, REM-sleep; NREM, NREM-sleep; Mvmt, movement; ND, not determined). For the detailed explanation of the applied calculations please refer to the Sections “Materials and Methods,” and “Electrophysiology Data Analysis.”

Video and local field potential recordings were started after 2–3 days of habituation to the recording home cage. Data were analyzed in 24 h long epochs. Local field potential recordings were filtered in the δ range (1–4 Hz) and the δ magnitude was calculated using the Hilbert transformation. Both activity values deriving from the video data and delta magnitudes were binned at 1 s bin width. The binned activity values were plotted against the binned delta magnitudes for a 24 h-long session. Based on the point clouds, 3 clusters were separated (low δ + high activity, low δ + low activity, high δ + low activity) and thresholds for δ and activity values between the clusters were determined (**Figure 1A**).

Using these thresholds a custom made software (written in Igor64, Wavemetrics, Lake Oswego, OR, USA) categorized every second of the long local field potential recording into one of the following 4 groups: movement (activity + low δ), NREM sleep (zero activity + high δ), REM sleep (zero activity + low δ) and a fourth category which consisted of segments that could not be determined (ND; **Figure 1B**).

**FIGURE 2:**
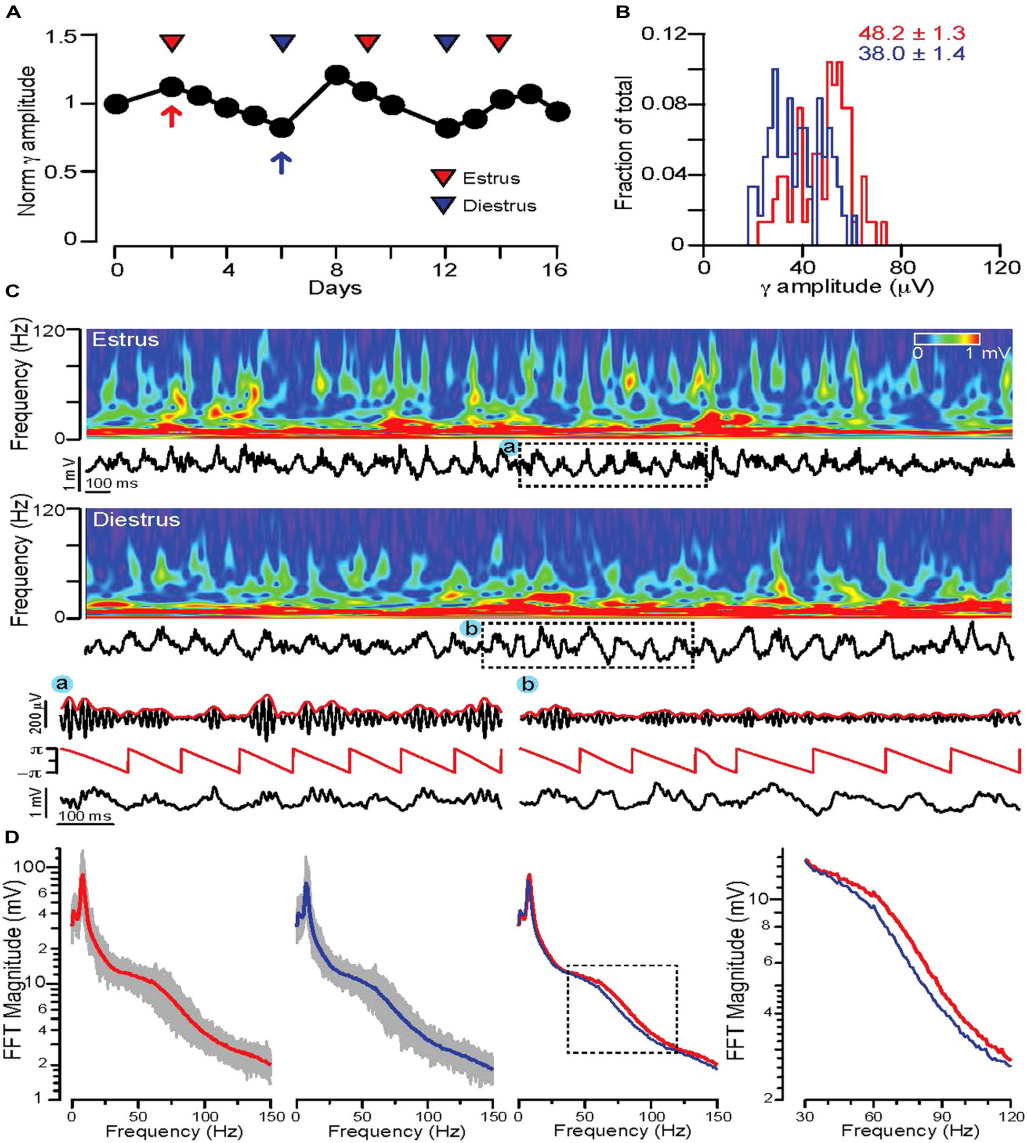
**Fluctuations in REM sleep γ oscillation magnitudes correlate with stages of the ovarian cycle in WT mice. (A)** Diagram showing average normalized 𝜃 phase coupled γ amplitudes during REM sleep on consecutive days in a WT cycling female mouse. Red and blue triangles indicate estrus and diestrus days, respectively. **(B)** Distribution of γ amplitudes (binned at 2 μV) recorded in all REM phases of a single day of estrus (red) and one of diestrus (blue). The same days are indicated on **(A)** with red (estrus) and blue (diestrus) arrows. Note the shift of the distribution toward larger values during estrus. Colored numbers indicate mean ± SEM of the corresponding histograms. **(C)** Top rows: 4 s long local field potential recordings with the corresponding wavelet spectra during REM sleep of estrus (top) and diestrus (bottom). **(A)** and **(B)**: 𝜃 phase and γ magnitude components of local field potential segments indicated by dashed rectangles. *Below*: Hilbert phases of the 𝜃 (5–12 Hz) frequency range, filtered trace in the γ frequency range (63–120 Hz, black) with the corresponding Hilbert magnitudes (red). **(D)** FFT spectra of local field potential recordings of all REM phases during an estrus (leftmost diagram) and a diestrus day (second from left). Gray traces indicate the FFT spectra of individual REM phases, thick lines are the average FFT spectra on an estrus (red) or diestrus (blue) day. Third diagram from left shows the superimposed two average FFT spectra (estrus: red, diestrus: blue) for comparison. The area marked by dotted lines is enlarged on the diagram on the right to show the segments of the average FFT spectra where the largest deviation appears in γ activity. For the detailed explanation of the applied calculations please refer to the Sections “Materials and Methods,” and “Electrophysiology Data Analysis.”

**FIGURE 3:**
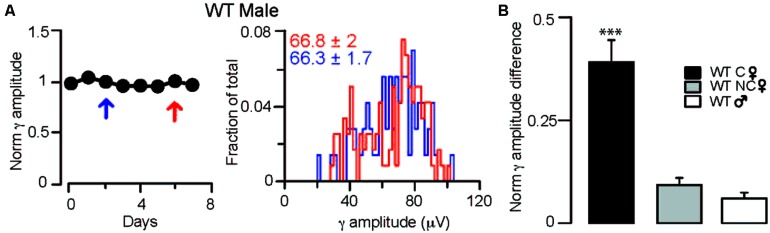
**Fluctuations in γ oscillation amplitudes recorded during REM sleep are absent in males and non-cycling WT females. (A)** *Left*: diagram showing normalized γ amplitudes during REM sleep on consecutive days in a WT male mouse. *Right*: distribution of γ amplitudes (binned at 2 μV) in all REM phases recorded on two separate measurements taken 3 days apart. The 2 days are indicated on the left with red and blue arrows. Colored numbers indicate mean ± SEM of the corresponding γ amplitude histograms. **(B)** Summary data showing the absolute values of the differences between the normalized γ amplitudes during REM sleep in different groups of mice. In the cycling WT females the difference was calculated between days of diestrus and estrus. In WT males and non-cycling females the differences in γ amplitudes were determined at 3-day intervals that approximated the time difference between the estrus and diestrus of cycling female mice. Asterisks indicate significant difference from all other groups in a Tukey’s multiple comparison test following a one-way ANOVA.

The definition of REM sleep was further narrowed by accepting only zero activity + low δ segments longer than 20 s that were adjacent to a segment characterized by high δ + zero activity (putative NREM sleep) phase. The detected REM segments were filtered in the 𝜃 (5–12 Hz) and high γ (63–120 Hz) range and 𝜃 phases and γ magnitudes were calculated using Hilbert transforms (**Figure 2C**). 𝜃 phase coupled γ amplitudes were determined by calculating the difference between the min and max values of the 𝜃 phase triggered average γ magnitude. On **Figures 2, 3**, and **5** the γ amplitudes were normalized to the mean values across all days and then plotted as a function of days. For male and non-cycling female mice the differences in γ amplitudes were determined at 3 day intervals that approximated the time difference between the estrus and diestrus phases of cycling female mice.

Time-frequency representation of the signal (**Figure 2C**) was performed using the Morlet wavelet transform. The magnitude of the wavelet transform was plotted as a function of time and frequency, with warmer colors representing increasing magnitude.

### STATISTICS

Data are expressed as mean ± SEM. For group comparisons we used one-way ANOVA with Tukey’s *post hoc* test corrected for multiple comparisons. *p* < 0.05 were accepted as significant differences.

## RESULTS

### THE AMPLITUDE OF HIPPOCAMPAL γ OSCILLATIONS RECORDED *IN VIVO* FLUCTUATES WITH PHASES OF THE OVARIAN CYCLE

In light of current evidence about cognitive performance fluctuations over the ovarian cycle in both women and rodents, it is remarkable that γ oscillations, a network activity that has been implicated in memory and encoding (Singer, 1993; Colgin and Moser, 2010; Uhlhaas et al., 2011; Buzsáki and Wang, 2012), have not been examined in relationship to the menstrual cycle in women or to the ovarian cycle in rodents. We therefore sought to measure γ oscillations coupled to 𝜃 rhythms in cycling female WT mice. We focused on REM 𝜃 – γ episodes, because this state is relatively easy to identify, shows consistent 𝜃 phase γ amlpitude coupling and appears to be linked to emotional information processing and memory consolidation (Montgomery et al., 2008; Walker, 2009; Scheffzük et al., 2011).

To test possible ovarian cycle related changes in γ activity during REM 𝜃-γ episodes, in the first set of experiments continuous video local field potential recordings were performed for 2–3 weeks in cycling female WT mice. REM phases were detected and the average 𝜃 phase coupled γ amplitudes were calculated for each consecutive day. Plotting the normalized γ amplitudes revealed a characteristic fluctuation, which correlated with the stage of the ovarian cycle determined by vaginal impedance or cytology (**Figure 2A**).

Comparing the distribution of γ amplitudes for a large number (*n* > 10/day) of REM segments in estrus and diestrus indicated a shift toward higher γ amplitudes during estrus (averages of normalized γ amplitudes across animals: 1.21 ± 0.04 for estrus, 0.82 ± 0.04 for diestrus, *n* = 2 mice). Comparing the wavelet spectrogram of sample REM segments of a representative estrus and a diestrus day, revealed a more prominent presence of higher γ frequencies during estrus (**Figure 2C**). The average FFT spectra of all REM phases during an estrus and diestrus day showed a clear shift toward higher γ frequencies (**Figure 2D**). To investigate this alteration in γ oscillations throughout the study we focused on the 𝜃 phase coupled γ activity (γ amplitude) in the frequency range (63–120 Hz) where the largest shifts were found in the FFT spectra.

### THE AMPLITUDE OF γ OSCILLATIONS IS CONSTANT IN WT MALE AND NON-CYCLING FEMALE MICE

To ensure that the dependence of the observed γ rhythm fluctuations on the stages of the ovarian cycle was not a random phenomenon, we also investigated possible alterations in γ oscillation magnitude over several days in WT males (averages of normalized γ amplitudes across animals: 1.00 ± 0.01, 0.98 ± 0.01 for the first and last days of a shifting 3-day window, *n* = 2 mice; **Figure 3B**) and non-cycling females (averages of normalized γ amplitudes across animals: 1.02 ± 0.02, 0.98 ± 0.03 for the first and last days of a shifting 3-day window, *n* = 2 mice; **Figure 3B**). During REM, 𝜃 coupled γ amplitudes did not reveal fluctuations over similar temporal windows in these 2 groups, demonstrating that in the absence of ovarian cycle-linked hormonal fluctuations there are no periodic changes in γ activity.

### THE OVARIAN CYCLE IS ASSOCIATED WITH δ-GABA_A_R SUBUNIT EXPRESSION CHANGES IN PRINCIPAL CELLS AND INTERNEURONS OF THE HIPPOCAMPUS

We have previously shown how γ oscillations dynamics *in vitro* are controlled by δ-GABA_A_Rs expressed on PV-INs (Mann and Mody, 2010) and plasticity of these receptors during pregnancy alters network excitability and γ oscillations frequency (Maguire et al., 2009; Ferando and Mody, 2013a). δ-GABA_A_Rs expression modulation during the ovarian cycle has been described in hippocampal DGGCs with direct consequences on the tonic GABA conductance, anxiety and cognitive performance (Maguire et al., 2005; Maguire and Mody, 2007; Cushman et al., 2014). Specifically, δ-GABA_A_Rs expression is decreased in the hippocampus of WT mice during estrus, when plasma progesterone levels are low. The tonic GABA conductance recorded in DGGCs is also decreased, and mice exhibit higher degrees of anxiety and trace fear conditioning during this stage of the ovarian cycle, indicating the functional nature of the observed GABA_A_R plasticity. In these studies, the plasticity of δ-GABA_A_Rs over the ovarian cycle has been demonstrated in whole hippocampal western blot analyses, and by immunohistochemistry in the periaqueductal gray region (Lovick et al., 2005). In the hippocampus, δ-GABA_A_Rs are expressed by most principal cells and some INs, and in both cell types they show high levels of plasticity during states of altered NS production (Maguire et al., 2009; Shen et al., 2010; Ferando and Mody, 2013a).

Here, in a broad manner, we addressed the hippocampal neuronal cell-type specificity of ovarian cycle-linked fluctuating expression of δ-GABA_A_Rs. With the use of δ-GABA_A_Rs specific antisera, we stained brains of WT female mice perfused at different stages of their ovarian cycle. The cycles were induced and determined as previously described (Maguire et al., 2005). In a separate staining we also compared δ-GABA_A_Rs expression in CA1 and CA3 SP-INs in cycling WT females to males and non-cycling WT females. All sections were processed in parallel to allow for accurate staining intensity comparisons.

In the hippocampus δ-GABA_A_Rs are found in the dendritic compartments of DGGCs and to a lesser extent in CA1 PCs, but not CA3 PCs. Moreover, δ-GABA_A_Rs are expressed by different types of IN, including neurogliaform cells of the DG and lacunosum molecular and CA3, CA1 and DG PV + INs (Ferando and Mody, 2013a,b; Yu et al., 2013). INs expressing δ-GABA_A_Rs with their somata located in the SP or within 30 μm around the SP have been shown to have over a 95% chance of being PV+ (Ferando and Mody, 2013a; Yu et al., 2013).

**FIGURE 4:**
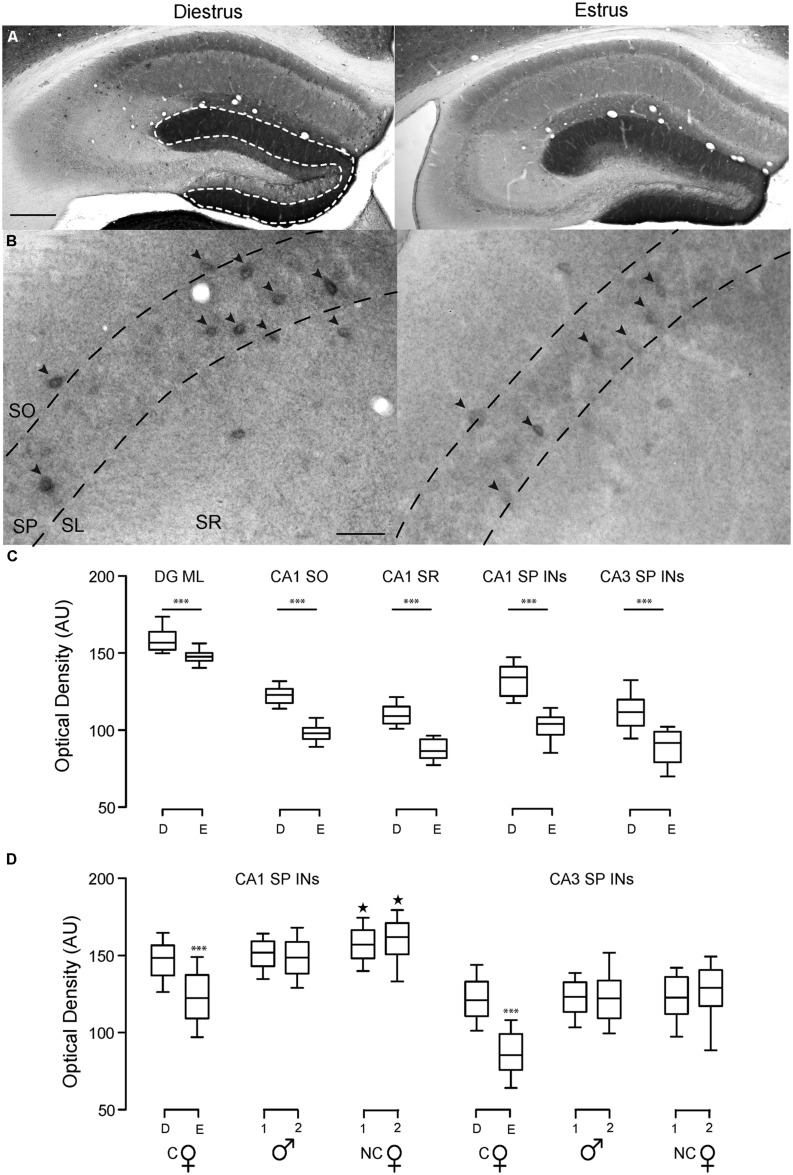
**Hippocampal δ-GABA_**A**_R plasticity at different stages of the ovarian cycle.** Representative bright field images of hippocampal DAB staining showing δ-GABA_A_R expression patterns in WT cycling females at different stages of the ovarian cycle. In the hippocampus, δ-GABA_A_Rs are heavily expressed in the molecular layer of the dentate gyrus (DGML) and on numerous INs including neurogliaform cells and PV-INs CA1 strata oriens and radiatum (CA1 SO and SR). Expression in CA1 PC dendrites in the SR is less prominent, but noticeable. **(A)** δ-GABA_A_R expression in DGGCs and CA1 pyramidal cells is lower in estrus compared to diestrus, and this is reflected in the staining intensity in the DGML and CA1 SO and SR. Scale bar 200 μm. **(B)** In CA1 and CA3 SP INs are strongly labeled with δ-GABA_A_R. Optical densities of δ-GABA_A_R expression were measured only in these INs (black arrowheads). Labeling of INs is weaker during estrus, which suggests lower δ-GABA_A_Rs expression on SP-INs (95% of which are PV-INs) during this stage of the ovarian cycle. Scale bar 50 μm. **(C)** Optical density measurements are in AU. Box plots represent the 25th and 75th percentile, the line in the middle is the median, and the 10th and 90th percentile are indicated by the error bars. ***p < 0.0001 difference from all other groups. **(D)** Optical density measurements of CA1 and CA3 SP INs in slices of cycling WT female mice (C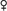) in diestrus (D) or estrus (E), male mice (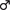) and non-cycling WT female mice (NC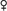). δ-GABA_A_Rs expression on SP-INs during estrus is lower than any other group in both CA1 and CA3 (***p < 0.0001); in the CA1 the two NC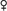 groups are both higher than any other group in CA1 (^∗^*p* < 0.0001). Significance levels were established by one-way ANOVA followed by Tukey’s multiple comparisons test. All sections for the separate measurements in **(C)** and **(D)** were processed together to allow for densitometric comparisons.

We found that δ-GABA_A_Rs expression fluctuates over the ovarian cycle in DGGCs, CA1 PCs, and CA1 and CA3 SP INs (**Figures 4A–C; Table 1**). In particular, during times of low plasma progesterone (estrus), staining for δ-GABA_A_Rs is significantly lower compared to times of high plasma progesterone (diestrus), which is suggestive of a downregulation of δ-GABA_A_Rs expression during estrus. We found that δ-GABA_A_Rs expression on CA1 and CA3 SP-INs is similar between diestrus female and male mice, while non-cycling females have a slightly increased expression selectively in CA1 SP-INs (**Figure 4D**; **Table 1**).

### OVARIAN CYCLE-LINKED FLUCTUATIONS IN γ OSCILLATIONS AMPLITUDES DEPEND ON THE PRESENCE OF δ-GABA_A_Rs ON PV-INs

Since more than 95% of SP INs that express δ-GABA_A_Rs also express PV (Ferando and Mody, 2013a), the observed plasticity in δ-GABA_A_Rs through the ovarian cycle is likely to influence the functioning of PV-INs. Oscillations induced in brain slices in the γ frequency have been shown to be controlled by δ-GABA_A_Rs of INs (Mann and Mody, 2010).

**FIGURE 5:**
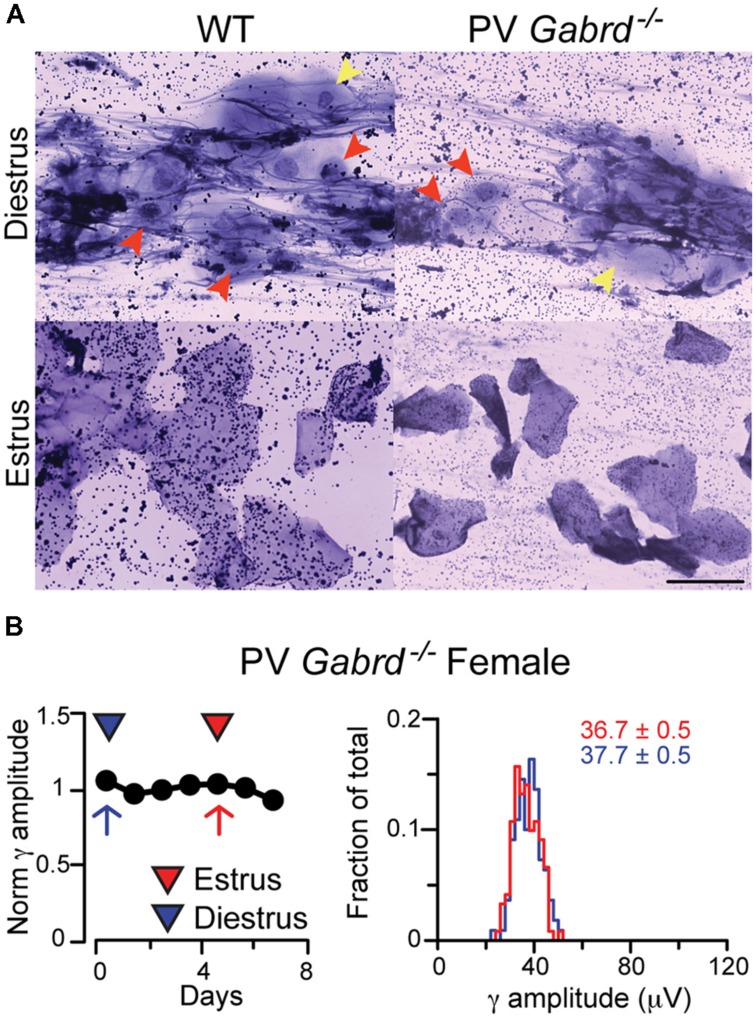
**Female PV-*Gabrd^-/-^* mice cycle regularly but lack ovarian cycle-dependent modulation of REM phase γ oscillation amplitudes. (A)** Ovarian cycle stage was determined by vaginal impedance measurements and cytological analysis of vaginal smears. PV-*Gabrd^-/-^* mice had a similar cytological panel to WT mice at different stages. Diestrus was determined by the presence in the smear of abundant mucus, small parabasal cells (red arrowheads) and large intermediate cells (yellow arrowheads). Both cell types are absent in estrus, when smears are typically characterized by large polygonal superficial cells, mostly fully cornified. Scale bar 5 μm. **(B)** Left: diagram showing normalized γ amplitudes during REM sleep on consecutive days in a regularly cycling PV-*Gabrd^-/-^* female. Red and blue triangles indicate estrus and diestrus, respectively. *Right*: distribution of γ amplitudes (binned at 2 μV) recorded during all REM sleep episodes for an entire day of estrus (red) and one of diestrus (blue). The 2 days are marked on the left with red (estrus) and blue (diestrus) arrows. Colored numbers indicate mean ± SEM of the corresponding histograms. Note the narrow variance of the γ amplitude distributions during both phases of the ovarian cycle.

In order to address possible functional correlates to the observed δ-GABA_A_Rs plasticity on CA1 and CA3 SP INs, we generated mice that lack the δ subunit of the GABA_A_R selectively in PV + INs. These mice lose the great majority of δ-GABA_A_Rs staining in CA1 and CA3 SP and its close proximity (within 30 μm), which confirms the previously described preferential distribution of δ-GABA_A_Rs on PV-INs in these areas (Ferando and Mody, in preparation, data not shown). However, the mice have normal ovarian cycles as indicated by the vaginal smears of WT and PV-*Gabrd^-/-^* mice in diestrus and estrus. When induced with litter carrying the smell of male urine, PV-*Gabrd^-/-^* females exhibit regular ovarian cycling that can be ascertained with the use of both vaginal impedance measurements and cytological analysis of vaginal smears (Maguire et al., 2005). Their smears are indistinguishable from those of WT mice; i.e., the diestrus phase is characterized by small parabasal cells, large intermediate cells and abundant mucus, while estrus is characterized by large cornified anucleated superficial cells (**Figure 5A**).

Once we established that PV-*Gabrd^-/-^* females have regular ovarian cycles, we went on to measure γ oscillations coupled to 𝜃 rhythms during REM sleep periods during the estrus and diestrus stages of the ovarian cycle, as we have done for WT females. Interestingly, we could not observe any fluctuations in γ oscillation magnitude in cycling PV-*Gabrd^-/-^* female mice (averages of normalized γ amplitudes across animals: 1.02 ± 0.04 for estrus, 0.97 ± 0.03 for diestrus, *n* = 2 mice) indicating the requirement of intact δ-GABA_A_Rs on PV + INs for the observed fluctuations in γ amplitudes. Comparing the difference in γ amplitudes calculated between diestrus and estrus or between measurements taken 3 days apart (for explanation, see Materials and Methods) in the 4 groups revealed significant fluctuation in the γ amplitudes in cycling WT mice (based on 6 estrus – diestrus days for cycling WT and 4 estrus – diestrus days for cycling PV-*Gabrd^-/-^* mice, 2 animals in each group *F*_(3,21)_ = 3.072, *p* < 0.0001).

## DISCUSSION

In this study we describe fluctuations of γ oscillation amplitudes recorded during REM sleep *in vivo* that are coupled to distinct phases of the ovarian cycle. Such periodic fluctuations in γ oscillation amplitudes were not present in male or non-cycling female mice. The γ amplitude fluctuations are inversely related to the level of expression of δ-GABA_A_Rs hippocampal INs located in the SP, and critically depend on the presence of δ-GABA_A_Rs on PV-INs. Although the broad shape of oscillation frequency spectra recorded *in vivo* makes it difficult to detect a precise shift in a single coherent frequency peak, we nevertheless noted a shift to the right of the recorded spectra during estrus in WT females, so that higher frequencies became more powerful. This finding is consistent with previous *in vitro* studies describing higher γ oscillations frequency during periods of low δ-GABA_A_Rs expression on PV-INs (Mann and Mody, 2010; Ferando and Mody, 2013a). Our findings are also consistent with our previous *in vivo* results showing increased γ oscillatory power in the olfactory bulb, after selective ablation of GABA_A_Rs on INs (Nusser et al., 2001).

As NS fluctuate over the cycle, so does the expression of the highly NS sensitive δ-GABA_A_Rs in different neurons of the hippocampus. Interestingly, levels of δ-GABA_A_Rs in hippocampal SP-INs at diestrus are comparable to those found in male mice, whereas δ-GABA_A_Rs expression decreases during estrus. Non-cycling females appear to have slightly higher δ-GABA_A_Rs levels than males, selectively on CA1 SP INs. Specific genetic and optogenetic manipulations of PV-INs have cemented their role in the local generation of γ oscillations (Sohal et al., 2009; Wulff et al., 2009; Korotkova et al., 2010; Carlén et al., 2011; Zheng et al., 2011; Lasztoczi and Klausberger, 2014). In line with these findings, our observations merely point out that hippocampal γ oscillation magnitude also depends on the expression of δ-GABA_A_Rs on these neurons. These receptors are extremely plastic, dramatically changing their expression levels within a few days during hormonal alterations of the ovarian cycle. The precise consequences of fluctuating γ oscillations on memory and cognitive performances may not always be easily predictable, although reports suggest enhanced memory performance to be correlated with higher γ frequency band magnitude (Lu et al., 2011).

A prediction based on our studies would be that cognitive processes in females would be enhanced during the low progesterone phase of the ovarian cycle (estrus) when γ oscillations are increased. Indeed, several studies reported higher hippocampus-mediated learning and memory performance and increased anxiety levels in female rodents during the estrus phase of the ovarian cycle, whereas animals in diestrus performed similar to males (Maguire et al., 2005; Walf et al., 2006; Moore et al., 2010; van Goethem et al., 2012; Cushman et al., 2014). Although at present it is unknown whether similar alterations in PV-IN δ-GABA_A_Rs also take place in humans, menstrual cycle-dependent variations in memory performance are not uncommon (Bayer et al., 2014). It also remains to be determined whether a higher cognitive capacity during the preovulatory phase may provide any evolutionary advantage.

In addition to INs, δ-GABA_A_Rs are also expressed on most principal cells of the hippocampus (Sperk et al., 1997; Glykys et al., 2007; Milenkovic et al., 2013; Ferando and Mody, 2013a). Although δ-GABA_A_Rs expression on CA1 PCs is modulated across the ovarian cycle (**Figure 4C**), this does not seem to result in appreciable changes in network level activity, as also supported by previous studies reporting comparable CA1 PC tonic conductances in diestrus and estrus (Maguire et al., 2005). This is not surprising as in these cells 70% of the total tonic inhibition is mediated by α5-GABA_A_Rs, which have been shown to easily compensate for δ-GABA_A_Rs loss (Glykys et al., 2008). In contrast, the tonic GABA conductance of hippocampal INs seems to be solely mediated by δ-GABA_A_Rs (Glykys et al., 2008). It is interesting to note the narrow variance of γ oscillation amplitudes in PV-*Gabrd^-/-^* mice. This phenomenon will need further investigation, as it is possible that complete lack or insufficient levels of δ-GABA_A_Rs on PV-INs may allow for restricted degrees of modulation of the γ oscillatory amplitudes, resulting in potentially disruptive effects on cognitive function. Unfortunately, our study using simple single site recordings does not permit accurate comparisons of the γ oscillation amplitudes across animals. Future multi-site recordings and current source density analyses will be required to ascertain any potential regional differences in the magnitude of γ oscillations between WT and PV-*Gabrd^-/-^* animals.

The molecular mechanisms responsible for δ-GABA_A_Rs plasticity during the ovarian cycle, or following steroid fluctuations in general, are unknown but may involve protein phosphorylation, and transcriptional modifications (Choi et al., 2008; Jacob et al., 2008; Abramian et al., 2010; Saliba et al., 2012). Recently, intracellular Cl^-^ itself has been proposed to function as a messenger for plasticity of different GABA_A_Rs subunits (Succol et al., 2012). Nonetheless, NS synthesis is a necessary event for δ-GABA_A_Rs modulation over the ovarian cycle (Maguire and Mody, 2007).

The lack of γ oscillation modulation in PV-*Gabrd^-/-^ in vivo* suggests that pathological alteration in the normal phsyiology of IN-δ-GABA_A_Rs through the ovarian cycle may have important consequences on how information is processed at the network level, and may predispose to pathological conditions if combined with aggravating events that lead to altered NS production or inadequate IN δ-GABA_A_Rs plasticity. Therefore, the development of δ-GABA_A_Rs specific drugs to selectively control IN function may be a novel future approach to the treatment of these symptoms in women with premenstrual syndrome (PMS) and PMDD.

## AUTHOR CONTRIBUTIONS

Albert M. I. Barth, Isabella Ferando, and Istvan Mody designed research; Albert M. I. Barth performed electrophysiology experiments; Isabella Ferando performed immunohistochemistry experiments and determined the ovarian cycle stages; Albert M. I. Barth, Isabella Ferando, and Istvan Mody analyzed data; Isabella Ferando and Istvan Mody wrote the paper.

## Conflict of Interest Statement

The authors declare that the research was conducted in the absence of any commercial or financial relationships that could be construed as a potential conflict of interest.
